# Development of a Three-dimensional Surgical Navigation System with Magnetic Resonance Angiography and a Three-dimensional Printer for Robot-assisted Radical Prostatectomy

**DOI:** 10.7759/cureus.2018

**Published:** 2018-01-02

**Authors:** Wataru Jomoto, Masao Tanooka, Hiroshi Doi, Keisuke Kikuchi, Chiemi Mitsuie, Yusuke Yamada, Toru Suzuki, Toshiko Yamano, Reiichi Ishikura, Noriko Kotoura, Shingo Yamamoto

**Affiliations:** 1 Department of Radiological Technology, Hyogo College of Medicine College Hospital; 2 Department of Radiology, Hyogo College of Medicine; 3 Department of Urology, Hyogo College of Medicine

**Keywords:** robotic surgical procedures/is, magnetic resonance imaging (mri), three-dimensional printing, magnetic resonance angiography

## Abstract

We sought to develop a surgical navigation system using magnetic resonance angiography (MRA) and a three-dimensional (3D) printer for robot-assisted radical prostatectomy (RARP). Six patients with pathologically proven localized prostate cancer were prospectively enrolled in this study. Prostate magnetic resonance imaging (MRI), consisting of T2-weighted sampling perfection with application-optimized contrasts using different flip-angle evolutions (SPACE) and true fast imaging with steady-state precession (true FISP), reconstructed by volume rendering, was followed by dynamic contrast-enhanced MRA performed with a volumetric interpolated breath-hold examination (VIBE) during intravenous bolus injection of gadobutrol. Images of arterial and venous phases were acquired over approximately 210 seconds. Selected images were sent to a workstation for generation of 3D volume-rendered images and standard triangulated language (STL) files for 3D print construction. The neurovascular bundles (NVBs) were found in sequence on non-contrast images. Accessory pudendal arteries (APAs) were found in all cases in the arterial phase of contrast enhancement but were ill-defined on non-contrast enhanced MRA. Dynamic contrast-enhanced MRA helped to detect APAs, suggesting that this 3D system using MRI will be useful in RARP.

## Introduction

Prostate cancer is the most prevalent of all cancers in men in Western nations. The incidence and prevalence of prostate cancer have risen steadily across the globe, accounting for 28% and 22.2% of new cancer diagnoses in American men in 2010 and European men in 2008, respectively [1]. In Japan, prostate cancer has also steadily increased and is now the most frequently diagnosed cancer in men. Global temporal trends in the incidence of prostate cancer are, in part, a reflection of the widespread use and effectiveness of prostate-specific antigen (PSA) screening tests. Walsh, et al. has reported on the refinement of radical prostatectomy (RP) to include nerve-sparing procedures, which has resulted in a reduction in the incidence of postoperative erectile dysfunction [2-3].

Robot-assisted radical prostatectomy (RARP) was first conducted using the da Vinci system in 2000. Since then, RARP has rapidly become the preferred surgical procedure for clinically localized prostate cancer across the globe [4]. While RARP was initially introduced to Japan in 2006, its use was limited because it was not covered by the Japanese health insurance system. However, in April 2012, the Japanese Ministry of Health, Labor, and Welfare agreed to cover RARP procedures under its health insurance scheme, leading to a surge in RARP use. This robotic equipment simultaneously improves visualization and precision and allows the surgeon to resect the cancer with a lower incidence of adverse effects, such as urinary incontinence and erectile dysfunction.

While the gold-standard imaging modality for assessment of vessels is multidetector-row computed tomographic angiography (CTA) [5], Whang, et al. have reported the utility of dynamic contrast-enhanced magnetic resonance angiography (MRA) in order to evaluate the vessels surrounding the prostate [6]. In addition, improvements in magnetic resonance imaging (MRI) technology have enabled the provision of spatial information for surgical planning. MRI is also useful for clinical staging and for determining the anatomical features of the prostate and bony pelvic dimensions. Despite several reports on three-dimensional (3D) MRI in head and neck surgery, plastic surgery, and orthopedic surgery [7-8], only a limited number of reports have dealt with sequences best able to provide significant information for the performance of RARP [9-10].

In this study, we aimed to develop a surgical navigation system using MRI for RARP.

## Technical report

Materials and methods

Patient Selection and Clinical Characteristics

This prospective study was approved by the institutional review board (approval number: 2272), and informed consent was obtained. All procedures performed in studies involving human participants were in accordance with the ethical standards of the institutional committee and with the 1964 Helsinki Declaration and its later amendments or comparable ethical standards. In this single-institution study, between August 2016 and January 2017, six patients scheduled for RARP were enrolled (Table 1). The inclusion criterion was a definitive diagnosis of prostate adenocarcinoma. Exclusion criteria were contraindications to MR examination or use of gadolinium-based contrast medium. MRI was performed immediately after micturition. It was followed by MR examination.

**Table 1:** Summary of findings of all cases PSA: prostate-specific antigen; MRI: magnetic resonance imaging

Case	Age (years old)	Clinical stage	Gleason score	Initial PSA level (ng/ml)	Volume of prostate in MRI (ml)
1	64	cT2N0M0	3+4	11.8	68.8
2	62	cT2N0M0	3+3	5.39	34.2
3	72	cT2bN0M0	4+3	10.4	23.1
4	74	cT1cN0M0	3+3	4.80	87.0
5	67	pT2cN0M0	3+4	4.08	36.4
6	67	cT2aN0M0	3+4	7.18	40

MRI Acquisition Protocol

MRI was performed using a 3.0 T MR scanner (MAGNETOM Skyra, Siemens Healthineers, Erlangen, Germany) with a spine matrix coil and body matrix coil for signal reception. The sequences were created for navigation and differ from the diagnostic sequence described in the Prostate Imaging-Reporting and Data System (PI-RADS) [11]. All patients underwent a transverse T2-weighted volumetric MRI sequence (sampling perfection with application-optimized contrasts using different flip-angle evolutions (SPACE)) with TR = 1,800 ms, apparent TE = 83 ms, echo space = 3.78 ms, echo train length = 80, voxel size = 0.9 × 0.9 × 1.26 mm (reconstructed 0.45 × 0.45 × 0.7 mm), 630 Hz/pixel bandwidth, and a variable refocusing flip angle. Transverse 3-D true FISP (true fast imaging with steady-state precession) was performed with TR = 900 ms, TE = 1.96 ms, echo space = 3.9 ms, voxel size = 0.76 × 1.15 × 1.18 mm (reconstructed 0.57 × 0.57 × 0.6 mm), 840 Hz/pixel bandwidth, flip angle = 80°, and chemical shift selective (CHESS) fat suppression. A transverse T1-weighted VIBE (volume interpolated breath-hold examination) sequence, in combination with a parallel acquisition technique (integrated parallel acquisition technique: iPAT), was also acquired with TR = 3.71 ms, TE = 1.37 ms, voxel size = 0.98 × 0.98 × 2.00 mm (reconstructed 0.49 × 0.49 × 1.0 mm), 510 Hz/pixel bandwidth, flip angle = 20°, and quick fat-saturation mode (Q-fat sat). Subsequently, dynamic contrast-enhanced MRA was performed with a transverse T1-weighted VIBE sequence during intravenous bolus injection of gadoteridol (0.1 mmol/kg), with axial images of arterial and venous phases acquired over approximately 210 s.

3D Workstation and 3D Model

All MR data were sent to a workstation (ZIOsoft, Inc., Tokyo, Japan). Volume rendered (VR) images were created using T2-SPACE, True-FISP, and T1-weighted VIBE with contrast enhancement. Images of the periprostatic blood vessels were generated using True-FISP and T1-weighted VIBE. Standard triangulated language (STL) files were created for 3D printing using a MUTOH personal 3D printer MF 2000 (Mutoh, Inc., Osaka, Japan).

Results

**Figure 1:**
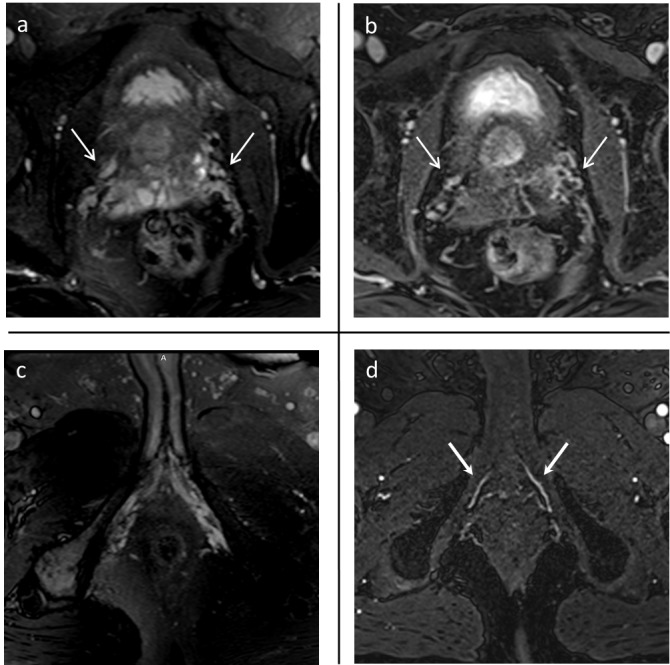
MR images in a 67-year-old man with prostate carcinoma (Case 6) Axial true-FISP (a) and T1-weighted VIBE contrast-enhanced images (b) show neurovascular bundles (NVBs) (arrows). The accessory pudendal arteries (APAs) are not visible on axial true-FISP (c) but brightly enhance on T1-weighted VIBE contrast-enhanced images (d) (arrows). Axial true-FISP (a, c) are non-contrast-enhanced images. The T1-weighted VIBE contrast-enhanced image (b) is the venous phase. The T1-weighted VIBE contrast-enhanced image (d) is the arterial phase after 30 seconds of dynamic. In axial true-FISP and T1-weighted VIBE contrast-enhanced images, the depiction of NVB was good, but the depiction of APA was superior in the T1-weighted VIBE contrast-enhanced image.

Figure 1 shows images of a 67-year-old male with prostate adenocarcinoma, cT2aN0M0, an initial prostate-specific antigen (iPSA) level of 7.18 ng/mL, and a Gleason score 3 + 4 (Case 6). The neurovascular bundles (NVBs) are visualized on unenhanced and enhanced images and the APAs on enhanced VIBE images.

**Figure 2:**
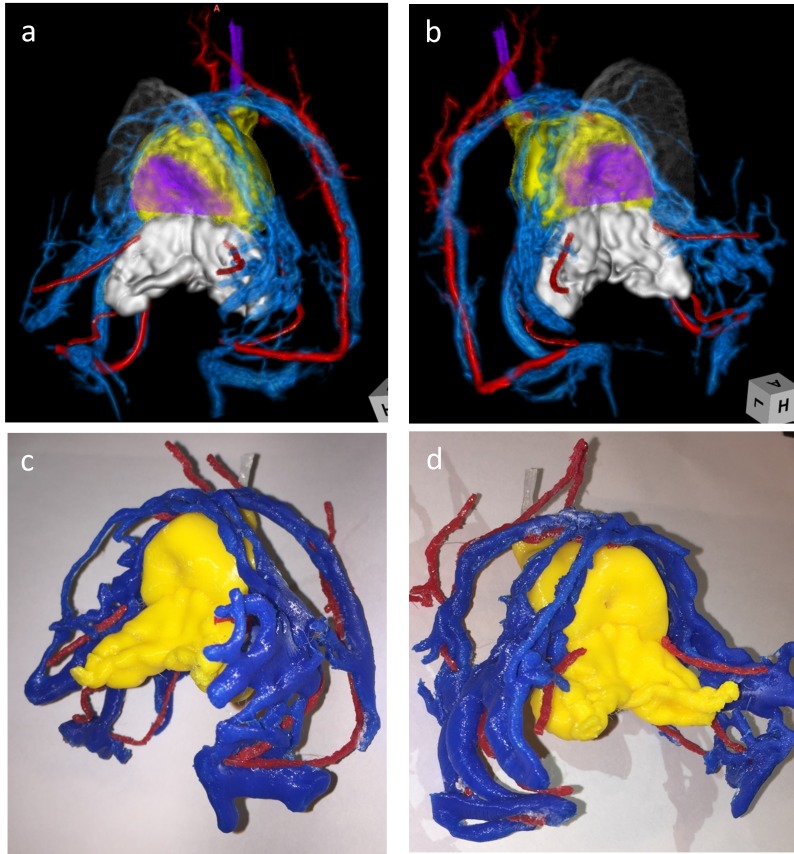
Three dimensional (3D) model of a typical case (Case 6) Volume rendered (VR) images (a, b) were created using T2 SPACE and true-FISP and contrast-enhanced T1-weighted VIBE. The soft tissues, such as prostate and seminal vesicles, were volume rendered using T2 SPACE. The representation of periprostatic blood vessels was generated using true-FISP and T1-weighted VIBE. The VR images were generated from standard triangulated language (STL) files constructed for 3D printing. Arteries in each image are represented in red and the veins in blue.

The NVBs were detectable in five patients on non-contrast-enhanced MRI scans and in one patient on a dynamic contrast-enhanced MRI. The accessory pudendal arteries (APAs) were detectable in all patients on dynamic contrast-enhanced MRI and in three patients on non-contrast-enhanced sequences. Identification of NVBs and APAs on MRI is shown in Table 2. A 3D model of Case 6 is shown in Figure 2.

**Table 2:** Identification of NVBs and APAs on MRI NVBs: neurovascular bundles; APAs: accessory pudendal arteries; MRI: magnetic resonance imaging; CE: contrast-enhanced; non-CE: non-contrast-enhanced

Case No.	NVBs	APAs
1	CE, non-CE	CE
2	CE, non-CE	CE, non-CE
3	CE, non-CE	CE
4	CE	CE
5	CE, non-CE	CE, non-CE
6	CE, non-CE	CE, non-CE

The images and 3D models of the bladder neck and NVBs were very useful and helped the surgeons to know the shape of the prostate and the course of the NVBs as they performed bladder neck dissection, as well as nerve sparing. However, it was impossible to identify the APAs intraoperatively in these cases. The vascularity of 3D models was overestimated, but the position of the blood vessel could be confirmed and visualized before the operation, which helped surgery.

Case 4 had a relatively large prostate. In two patients, the prostate protruded into the bladder (Cases 3 and 5). Case 2 had a history of transurethral resection of the prostate. Four patients required no urinary pads at six months after RARP. Two remaining patients wore one pad per day due to mild incontinence at three months after RARP. In addition, no patients developed severe complications related to imaging and RARP. In Cases 2 and 6, bilateral NVB preservation was planned. However, the erectile function of Cases 2 and 6 was already abnormal before RARP, and it had not recovered six months after surgery.

## Discussion

RARP is a widely accepted surgical approach for the treatment of prostate cancer. Due to advances in optics, video technology, and specialized instrumentation, the use of RARP has the advantage of minimal invasiveness while clearly displaying the periprostatic anatomy. The use of video-endoscopy allows better preservation of the structures around the urinary sphincter, such as APAs, improved apical dissection, and preservation of the NVBs [12-13]

The proportion of men with APAs varies according to the method used to detect them. Incidence has ranged from 4%–75% in radiological and pathological studies [6, 13-18]. In a retrospective clinical study, Park, et al. suggested a possible connection between APA preservation and postsurgical functional outcome [5]. The radiology literature describing the identification of APAs with incidence and anatomy is limited, though they have been described on CT images [17, 19]. However, MRI involves no radiation and can provide more accurate information for the determination of pelvic soft tissues. In this study, we used a spine matrix coil and body matrix coil. In daily clinical practice, MRI can be used more easily with a phased-array coil than with special coils, such as a transrectal coil.

Non-contrast MRA has a longer imaging time than dynamic contrast-enhanced MRA and is inferior to dynamic MRA in the detection of NVBs and APAs due to the influence of motion artifact due to rectal peristalsis. In our study, the NVBs and APAs were detected on dynamic MRI in all six patients. Dynamic MRI has been widely used to detect prostate tumors and to evaluate tumor stage [11]. Furthermore, fusing dynamic MRA and SPACE and constructing VR and 3D models allows better visualization of periprostatic tissues. There are various risk factors for postoperative incontinence, but NVB preservation significantly prevents postoperative urinary incontinence [20]. The erectile function of the two patients had not recovered. However, the postoperative urinary incontinence never lowered their quality of life. We hypothesize that the present technique could provide significant information for RARP with high acceptability for patients.

This study has several limitations. First, we can’t identify APAs intraoperatively. Since 60% of APAs are the apical type, it is often impossible to confirm APAs during surgery [18]. However, we believe that tracing of APAs by MRI and 3D modeling is likely to provide more accurate information about the incidence and anatomical associations of APAs. Further investigation is needed to clarify it. Second, our study had no independent standard of reference for detection of tissues surrounding the prostate. Therefore, small branches of vessels might have been missed on radiological images. However, the main branches of NVBs and APAs were well-detected in our 3D model. In addition, the prospective design of this study means that selection bias was likely minimal and without effect on the conclusions. Moreover, we suggest that 3D-guidance using MRI and a 3D printer are useful tools, not only for urologists but also for physicians in training, medical students, and medical staff. Further clinical research to assess the clinical benefits of 3D guidance based on the presented data is ongoing.

## Conclusions

Preoperative dynamic contrast-enhanced MRA provided good surveillance of the tissues surrounding the prostate, the NVBs, and APAs with the use of a 3D model and is a useful diagnostic option that may improve potency outcomes when used with RARP.
